# Treatment Strategies for Congenital Diaphragmatic Hernia: Change Sometimes Comes Bearing Gifts

**DOI:** 10.3389/fped.2017.00195

**Published:** 2017-09-14

**Authors:** Francesco Morini, Kevin P. Lally, Pamela A. Lally, Rosa Maria Crisafulli, Irma Capolupo, Pietro Bagolan

**Affiliations:** ^1^Department of Medical and Surgical Neonatology, Bambino Gesù Children’s Hospital, IRCCS, Rome, Italy; ^2^Department of Pediatric Surgery, McGovern Medical School at the University of Texas Health Science Center at Houston, Children’s Memorial Hermann Hospital, Houston, TX, United States

**Keywords:** congenital diaphragmatic hernia, extracorporeal membrane oxygenation, inhaled nitric oxide, intensive care, minimal access surgery, outcome, surgery

## Abstract

**Objective:**

To report treatment strategies’ evolution and its impact on congenital diaphragmatic hernia (CDH) outcome.

**Design:**

Registry-based cohort study using the CDH Study Group database, 1995–2013.

**Setting:**

International multicenter database.

**Patients:**

CDH patients entered into the registry. Late presenters or patients with very incomplete data were excluded. Patients were divided into three Eras (1995–2000; 2001–2006; 2007–2013).

**Main outcome measures:**

Treatment strategies and outcomes. One-way ANOVA, X2 test, and X2 test for trend were used. A Sydak-adjusted *p* < 0.0027 was considered significant. Prevalence or mean (SE) are reported.

**Results:**

Patients: 8,603; included: 7,716; Era I: 2,146; Era II: 2,572; Era III: 2,998. From Era I to Era III, significant changes happened. Some severity indicators such as gestational age, prevalence of prenatal diagnosis, and inborn patients significantly worsened. Also, treatment strategies such as the use of prenatal steroids and inhaled nitric oxide, age at operation, prevalence of minimal access surgery, and the use of surfactant significantly changed. Finally, length of hospital stay became significantly longer and survival to discharge slightly but significantly improved, from 67.7 to 71.4% (*p* for trend 0.0019).

**Conclusion:**

Treatment strategies for patients registered since 1995 in the CDH Study Group significantly changed. Survival to discharge slightly but significantly improved.

“Change always comes bearing gifts” Price Prichett.

## Introduction

Congenital diaphragmatic hernia (CDH) was first described by Lazare Riviere in a postmortem examination of a 24-year-old male (1). In 1754, George Macaulay reported the first neonate with CDH, also in a necropsy finding in an infant who died from respiratory failure (2). Treatment successes for CDH appeared at the beginning of the 20th century (3), suggesting improved survival with early surgery (4). However, it was not until 1940, when Ladd and Gross reported 9 survivors out of 16 operated CDH patients (5), that surgery became an accepted treatment for CDH. In 1946, Gross also reported the first successful repair of a CDH in a newborn in the first 24 h of life (6). At that time, surgical correction of CDH was considered a surgical emergency and immediate repair was the mainstay of the treatment with 90–95% reported survival rates (7). Since then, the management of CDH has undergone many modifications, shifting from a surgical emergency to a physiologic one. Treatments such as extracorporeal membrane oxygenation (ECMO), high-frequency ventilation, inhaled nitric oxide (iNO), and minimal access surgery have been introduced and gained popularity. In the last 15–20 years, several single-institution studies have reported the impact of management changes on the outcome of CDH patients, with controversial results (8–13). The only study on treatment and outcomes changes in CDH, involving a large cohort of patients, was published in 2006 but was limited to patients with diaphragmatic agenesis and showed no significant improvements in the outcomes (14).

The aim of present study is to report the evolution of management strategies for all newborns with high-risk CDH over the last two decades and to evaluate any association with changes in outcome.

## Materials and Methods

The CDH Study Group was formed in 1995 to compile data on live born infants with CDH at participating institutions to assess therapies and outcomes. The CDH Study Group consists of tertiary referral centers, distributed over four continents, which voluntarily provide data to a central registry (Appendix). Data on all infants with CDH who are born at or transferred to a participating center are entered into the database. Data are collected prospectively on all live born patients with CDH in participating hospitals and include information on delivery and subsequent hospital care until death or hospital discharge. The data from the registry forms are entered into a Microsoft (Redmond, WA, USA) Access database and are crosschecked against the original form. Patient demographics, birth information, treatment received, and outcome are recorded.

Patients with “high-risk” CDH (prenatal diagnosis and/or symptoms within the first 6 h of life) included in the Registry until December 2013 are the object of present study and are divided in three Eras (1995–2000, 2001–2006, 2007–2013). The definition of the Eras was made “*a priori*” and established to have three Eras of comparable length. Since 1995, there have been four versions of the data collection tool. With each update, the CDH Study Group has attempted to streamline information to provide an accurate reflection of the practice among individual centers. With each revision, data deemed unnecessary were eliminated and new data points were added as a result of lessons learned from previous versions. For this study, we included data that were collected throughout all the study period. Late presenters or patients with very incomplete data were excluded from the study, like outborn patients who were not referred to one of the participating centers.

### Primary Outcome Measure

Survival to discharge (defined as discharge from the tertiary hospitals, either home or back to referring hospitals).

### Secondary Outcome Measures

Prevalence of repair, prevalence of oxygen dependency at 30 days of life, length of mechanical ventilation, length of hospital stay.

In addition, the following variables were extracted.

### Severity Indicators

Gestational age, birth weight, and prevalence of prenatal diagnosis, inborn status, chromosomal anomalies, and major associated cardiovascular malformations. Major cardiovascular anomalies included hypoplastic left heart syndrome, coarctation of the aorta, and tetralogy of Fallot. An isolated ventricular septal defect or atrial septal defect was considered minor. In addition, the probability of survival in the three Eras was calculated, using the CDH Study Group formula: probability of survival = 1 − 1/(1 + e^−X^) where −X = −5.024 + 0.9165(BW) + 0.4512(Apgar5) (15). BW is the birth weight in kilograms and Apgar5 is the 5-min Apgar score in the discrete ordinate range 0–10.

### Treatment Strategies

Use of prenatal steroids, surfactant and iNO administration and ECMO, prevalence of repair, age at repair, use of minimal access surgery (either laparoscopy or thoracoscopy), type of open surgical approach (laparotomy vs thoracotomy) and minimal access surgical approach (laparoscopy vs thoracoscopy), patch repair, and of chest tube insertion.

### Statistical Analysis

Data were analyzed using GraphPad Prism 5.0 Macintosh Version (GraphPad Software, San Diego, CA, USA; www.graphpad.com). One-way ANOVA was used to compare the three groups for continuous variables, Chi-squared test and Chi-squared test for trend were used for categorical variables. Results are given as mean (SE) or prevalence, as appropriate. As several null hypotheses were tested, a Sydak-adjusted significance level of 0.0027 was calculated to account for the increased possibility of type-I error. Two-sided *p*-values are reported.

The CDH Study Group registry was approved by the University of Texas-Houston Institutional Review Board (HSC-MS-03-223). Participating centers filed a Waiver of Consent for data submission or signed a Data Use Agreement for a Limited Dataset.

## Results

In the study period, 8,603 newborns with CDH were prospectively enrolled in the CDH Study Group Registry. Late presenters (288 patients) and patients with very incomplete data (698 patients) were excluded. Therefore, 7,617 patients were included in the analysis: 2,146 in Era I; 2,572 in Era II; and 2,899 in Era III.

### Primary Outcome Measures

Overall survival to discharge slightly but significantly improved from Era I to Era III (Table 1). When each Era was compared with each other (Era I vs Era II: *p* = 0.3581; Era II vs Era III: *p* = 0.0274; Era I vs Era III: *p* = 0.0023), there was a significant improvement in survival from Era I to Era III. Patients died at 18.2 (1.3) days in Era I, at 27.0 (1.8) days in Era II, and at 25.0 (1.4) days in Era III (*p* = 0.0002; *p* for trend = 0.0017). The impact of patients’ severity on survival was also analyzed. In patients with some defined risk factors (inborn status, chromosomal anomalies, major CVM), survival rate changed but not reaching statistical significance. This was not true for patients requiring a patch repair, where we found a significant change in survival rate from 68.1 to 76.9% (*p* < 0.0001; *p* for trend < 0.0001), while those not requiring a patch had a non-significant change in survival (from 94.5 to 96.5%; *p* = 0.0597; *p* = 0.0269).

**Table 1:** Survival data (overall and in patients with defined risk factors).

Variable	Era I	Era II	Era III	*p*	*p* for trend

	1999–2000	2001–2006	2007–2013		
	
	2,146 pts	2,572 pts	2,899 pts
Overall survival (%)	67.6	68.9	71.6	0.0064	0.0019
Survival of inborn pts (%)	60.6	61.3	66.3	0.0080	0.0046
Survival of pts with chrom. anom. (%)	29.7	25.9	41.8	0.0146	0.0247
Survival of pts with major CVM (%)	30.9	44.2	40.2	0.0416	0.1418
Survival of pts with patch repair (%)	68.1	71.5	76.9	<0.0001	<0.0001

*Results are mean (standard error) or prevalence, as appropriate*.

*CVM, cardiovascular malformations; chrom. anom, chromosomal anomalies; Pts, patients*.

*Sydak adjusted p–value <0.0027 was considered significant*.

### Secondary Outcome Measures

Among secondary outcomes, only length of hospital stay significantly changed from 44.9 (1.3) days in Era I to 53.2 (1.2) days in Era III (*p* < 0.0001, *p* for trend < 0.0001) (Table 2).

**Table 2:** Secondary outcome measures.

Variable	Era I	Era II	Era III	*p*	*p* for trend

	1999–2000	2001–2006	2007–2013		
	
	2,146 pts	2,572 pts	2,899 pts
Operated patients (%)	82.3	82.3	83.0	0.7462	0.5062
Oxygen dependency at 30 days (%)	40.2	42.7	44.4	0.0365	0.0106
Length of mechanical ventilation (days)	19.0 (0.8)	19.7 (0.7)	19.7 (0.4)	0.6588	0.4087
Length of hospital stay (days)	44.9 (1.3)	50.3 (1.2)	53.2 (1.2)	<0.0001	<0.0001

*Results are mean (SE) or prevalence, as appropriate*.

*Pts, patients*.

*Sydak-adjusted p-value <0.0027 was considered significant*.

### Severity Indicators

Gestational age significantly decreased, while prevalence of prenatal diagnosis and prevalence of inborn patients significantly increased from Era I to Era III (Table 3).

**Table 3:** Severity indicators.

Variable	Era I	Era II	Era III	*p*	*p* for trend

	1999–2000	2001–2006	2007–2013		
	
	2,146 pts	2,572 pts	2,899 pts
Gestational age (weeks)	37.9 (0.1)	37.6 (0.1)	37.5 (0.1)	<0.0001	<0.0001
Birth weight (grams)	2,990 (10)	2,950 (10)	2,940 (20)	0.0319	0.0157
Chromosomal anomalies (%)	5.2	4.2	5.5	0.1632	0.5436
Major CVM (%)	6.6	8.4	8.4	0.0360	0.0332
Prenatal diagnosis (%)	47.4	61.4	67.4	<0.0001	<0.0001
Inborn (%)	35.4	43.1	45.2	<0.0001	<0.0001
Predicted survival (%)	62.7 (0.5)	64.7 (0.5)	64.2 (0.4)	0.0123	0.0125

*Results are mean (SE) or prevalence, as appropriate*.

*CVM, cardiovascular malformations*.

*Sydak-adjusted p-value <0.0027 was considered significant*.

### Treatment Strategies

Prenatal steroids administration, iNO use, age at operation, patch closure, and use of minimal access surgery (that progressively shifted from laparoscopy to thoracoscopy) significantly rose (Table 4). Conversely, surfactant and ECMO use and chest tube placement significantly dropped.

**Table 4:** Treatment strategies.

Variable	Era I	Era II	Era III	*p*	*p* for trend

	1999–2000	2001–2006	2007–2013		
	
	2,146 pts	2,572 pts	2,899 pts
Prenatal steroids (%)	12.1	14.4	22.6	<0.0001	<0.0001
Surfactant (%)	29.3	28.9	15.6	<0.0001	<0.0001
Inhaled nitric oxide (%)	27.1	50.5	61.6	<0.0001	<0.0001
ECMO (%)	37.5	31.6	30.3	<0.0001	<0.0001
Age at operation (days)	6.1 (0.2)	7.3 (0.2)	7.7 (0.2)	<0.0001	<0.0001
MAS (%)	0.1	2.1	15.3	<0.0001	<0.0001
Open thoracic approach (%)	4.2	4.0	5.3	0.1010	0.0909
Thoracoscopy (%)	0.0	69.9	90.2	<0.0001	<0.0001
Patch (%)	46.7	51.4	54.2	<0.0001	<0.0001
Chest tube (%)	65.0	42.8	34.9	<0.0001	<0.0001

*Results are mean (SE) or prevalence, as appropriate*.

*ECMO, extracorporeal membrane oxygenation; MAS, minimal access surgery*.

*Sydak-adjusted p-value <0.0027 was considered significant*.

## Discussion

In an analysis of over 7,500 neonates with high-risk CDH prospectively enrolled from multiple Institutions worldwide during the last two decades, we found that treatment strategies significantly changed and survival to discharge improved, despite the severity remained stable.

In CDH patients, the use of prenatal steroids and surfactant is based on the opinion that their lung is immature. Both experimental and human studies showed conflicting results on surfactant components in CDH lungs (16–19). Clinically, some suggest benefits with surfactant administration, and propose its use as routine or rescue therapy (20, 21), while data published from the CDH Study Group show no significant advantage of surfactant use, both in term and preterm infants with CDH (22), conversely raising some concerns over its routine use (22). A potential alternative to postnatal surfactant administration is to induce lung maturation by prenatal steroids. The inverse trends of decreasing postnatal surfactant administration and increasing prenatal steroids use may be an attempt to enhance lung maturation without the risks of postnatal surfactant administration. Experimental studies suggest that prenatal steroids significantly improve both lung maturation and postnatal outcome in CDH (23, 24). However, clinical data do not confirm the benefit of prenatal steroids administration (11, 20) and a recent report from the CDH Study Group does not support their use in CDH (25). As prenatal steroids use is not devoid of complications (26), it is prudent to confine antenatal steroids treatment in infants between 24 and 34 weeks of gestation, if delivery is expected before 34 weeks (27, 28).

Extracorporeal membrane oxygenation use progressively dropped, especially between Era I and Era II. ECMO is a complex and expensive technique that allows the blood to be oxygenated outside the body obviating the need for gas exchange in the lungs and is a last resort therapy in patients not responding to maximal conventional therapy. Neonates with CDH represent a distinct subgroup that seems to benefit less from ECMO, with a disappointing 51% survival (29). A recent meta-analysis on both retrospective and randomized studies on ECMO use in CDH showed controversial results (30). The meta-analysis of retrospective studies suggested a significant benefit from ECMO, especially for the more severe patients. Only two randomized studies could be included in the meta-analysis, showing only short-term advantages. ECMO provides effective but short-term support for the respiratory failure associated with CDH. The use of ECMO allows time for reversing the persistent pulmonary hypertension, otherwise lethal, avoiding high volume and/or pressure ventilation, which is traumatic for the lungs. However, ECMO does not have an effect on pulmonary hypoplasia and will be ineffective in patients with truly lethal pulmonary hypoplasia. As the degree of pulmonary hypoplasia cannot be foreseen accurately, the problem remains of predicting appropriate use of ECMO.

During the study period, the use of iNO progressively increased despite three different randomized controlled trials showed no benefit in patients with CDH (31–33). Nitric oxide is an endothelial-derived vasodilator that, inhaled, has a selective effect on the pulmonary arteries, and bronchodilator and anti-inflammatory effects. A recent Cochrane systematic review showed that iNO use resulted in prompt improvement of oxygenation and reduced the need for ECMO in neonates with persistent pulmonary hypertension, excepting CDH patients (34). It was concluded that term or near term neonates with hypoxic respiratory failure unresponsive to other therapy should have a trial of iNO, excluding infants with CDH. The increase seen during the study period, despite the available evidence, cannot be due to lack of awareness of the evidence. Most probably, physicians’ motivation to normalize physiology prevails when faced with a hypoxic ventilated CDH baby. As this treatment is hugely expensive and potentially harmful (35), further studies should aim to evaluate its efficacy and standardize its application in order to optimize patient outcomes and ensure cost-effective practices.

During the study period, age at operation and minimal access surgery use progressively rose. Although no clear evidence exists favoring delayed surgery in CDH (36), it is common belief that preoperative stabilization may improve the patients’ outcome. The progressive increase of age at operation may relate to a longer time required for patients’ stabilization and suggests increased severity. The progressive increase of the proportion of prenatal diagnosis, inborn patients, and patch repair, which correlate with severity and outcome (37, 38), are in agreement with this interpretation. One reason for this increase in severity may be the increased accuracy of prenatal diagnosis. Outborn severely ill CDH patients may die before transport to a tertiary referral center and hence constitute a hidden mortality. More accurate/sensitive prenatal diagnosis results in a higher detection rate of larger defects, with stomach and/or liver in the chest, and in an increase of “*in utero*” transfer of severely affected patients to tertiary referral centers. This hypothesis fits well with the increasing prevalence of patch repair, a proxy of defect size, which is a robust indicator of severity as it correlates with both mortality and morbidity at discharge (37, 39, 40). In theory, the increase in prenatal ultrasound accuracy may also lead to an increase in pregnancy termination. However, the stability of the proportion of patients with major cardiovascular anomalies and chromosomal anomalies, which are major causes inducing to termination of pregnancy, suggests that patterns of termination of pregnancy did not change over the study period. The progressive and corresponding increase in inborn patients we found over the three Eras rather corroborates the hypothesis of growing “*in utero*” referral of patients with prenatal diagnosis. The gradual increase of length of stay also suggests a worsening population, probably due to more demanding associated morbidities, as length of mechanical ventilation did not change over the study period. On the other hand, the disease severity, as predicted by the CDH Study Group formula (15), seemed stable over the study period.

Minimal access surgical approach progressively gained popularity, with a complete shift from laparoscopy to thoracoscopy. With its progression in the pediatric population, this approach was extended also to CDH patients. The comparison of minimal access surgery with open surgery for CDH shows controversial results, and the former was associated with higher recurrence rates (41) and intraoperative hypercapnea and acidosis (42), calling into question its safety. Consequently, its use should be cautious in patients with high-risk CDH and be limited to the less severe cases.

The Survival rate slightly but significantly improved over the study period, particularly between Era I and Era III, with the trend being statistically significant, suggesting an improvement in CDH management. It is possible that the concomitant increase seen in the severity of patients enrolled in the CDHSG registry may have led to the limited improvement in survival recorded in this study.

However, bordering the analysis to those groups of patients recognized as more severe, the increase in survival rate is more “clinical” than “statistical.” Survival increase from 61 to 66% in inborn patients, from 30 to 42% in those with chromosomal anomalies, from 31 to 40% in patients with major CVM, and from 68 to 77% in those requiring a patch. In all except those requiring a patch repair, these differences (approximating 10% improvement) did not reach statistical significance, possibly due to the design of the statistical analysis where we set the significance level well below the commonly accepted *p* < 0.05. These data suggest that changes in CDH management may have had an impact especially in the more severely affected patients. The finding regarding the change in survival in patients requiring patch repair is in contrast with the previous report from the CDH Study Group on patients with diaphragmatic agenesis, which showed no improvements in terms of mortality, hospital stay, and morbidity (14). It is possible that surgeon’s indication for patch repair became more liberal with time, with its use also in patients who would have undergone primary repair in the earlier years. On the other hand, the previous study was “limited” to 10 years while present study has a longer time span, allowing more time for treatment changes to influence the outcomes. The history of CDH outcome reporting shows bipolar courses with cycles of enthusiasm and nihilism. The majority of the reports derive from single tertiary referral institutions. This may lead to case selection bias and the existence of a “hidden mortality,” which is difficult to quantify (43). Outcomes analyzed at a supra-Institution level show a slight but significant improvement in the long run. The improvements in outcomes have been in turn attributed to the introduction of new treatment modalities. However, randomized studies, failed to show any true impact of their use, singularly, on the outcomes of CDH patients (31–33, 44–46). Most of these studies were not specifically designed for CDH patients, analyzed only as relatively small subgroups. Therefore, it is possible that they were not powered enough to detect the effects of the investigated treatments, if present. An alternative explanation is that the single intervention examined was not able to cause significant changes in the outcome by itself. In our cohort, a combined effect of several interventions cannot be excluded. Additionally, it is possible that other strategies that we were not able to assess, because not recorded in the registry, may have had an impact on survival evolution. The several changes in treatment strategies that happened during the last two decades combined together may have led to the improvement in survival rate seen in high-risk CDH patients enrolled in the CDH Study Group registry.

The CDH Registry was formed to allow collection and analysis of data on treatment and outcomes of infants with CDH from a large number of centers. While registries have proven to be a good way to collect information on various disorders, care should be taken in interpreting the data. In addition, they allow the analysis only on collected data, and we have limited the analysis only to data collected all along the study period. For example, data on the use of gentle ventilation, which was an important change introduced in the 1990s (47), are not recorded in the Registry and some of the data used to assess severity such as observed to expected lung to head ratio, the presence of the liver in the chest, or data used in the Brindle’s clinical prediction rule (48), were recorded only during the third Era. This may cause some uncertainity in the interpretation of the severity of patients groups in the three Eras. On the other hand, despite its limitations, present study allows the description of changes over a very long period, on a huge number of patients, which would have been impossible without a tool such as the Registry. The database is observational and conclusions about therapies should be made cautiously. The data are collected from institutions that differ significantly in their patient referral base and their criteria for accepting an infant with CDH for admission. Criteria for “non-salvageable” patients vary between centers and some patients in the repaired group in one center may not have undergone operation in another. Many infants with severe cardiac and chromosomal anomalies did not undergo repair making the true impact of these factors difficult to determine. There are also variations in surgical practice that may determine differences in timing of surgery and whether a patch is used. Similarly, the definition of agenesis is likely to vary somewhat between centers. Finally, the definition of the Eras was not based on the commencement or change in frequency use of each single treatment analyzed. However, this study is not designed to define the impact of one single treatment on the outcome of CDH. Rather, this study describes how treatment strategies used in CDH patients changed in the last decades and if this change was accompanied by a change in their outcome. While the characteristics of the registry does not allow to define the effects of a single treatment, its international and supra-Institutional nature is ideal to describe the changes in treatment strategies and overall trends of the outcomes. The observations of such a registry can be considered valid because they are free from single Institutions biases and policies are not driven by superimposed protocols. In addition, the huge number of patients enrolled gives further strength to the study leading to reliable interpretations and meaningful conclusions.

## Conclusion

The analysis of over 7,500 high-risk CDH patients shows several changes in the treatment strategies over the study period. During the same period, survival rate slightly but significantly improved, despite the patients’ population remained stable. Most of these changes were adopted based on common opinion rather than on evidence. Despite none of the treatments introduced represents *per se* the quested “magic bullet” in high-risk CDH management, a combined effect may be responsible for the improvements recorded. Data on long-term outcomes are currently missing from the registry. However, they would deserve attention to define if changes in early management of CDH patients have an impact on their long-term prognosis.

## Author Contributions

FM conceptualized and designed the study, carried out the initial analyses, drafted, reviewed and revised the manuscript, and approved the final manuscript as submitted. KL designed the data collection instruments, conceptualized and designed the study, critically reviewed the manuscript, and approved the final manuscript as submitted. PL designed the data collection instruments, coordinated and supervised data collection, reviewed and revised the manuscript, and approved the final manuscript as submitted. RC and IC reviewed and revised the manuscript, critically reviewed the manuscript, and approved the final manuscript as submitted. PB conceptualized and designed the study, critically reviewed the manuscript, and approved the final manuscript as submitted. All authors agree to be accountable for all aspects of the work in ensuring that questions related to the accuracy or integrity of any part of the work are appropriately investigated and resolved.

## Conflict of Interest Statement

The authors declare that the research was conducted in the absence of any commercial or financial relationships that could be construed as a potential conflict of interest.

## Appendix

**Table A1:** Tertiary referral centers that voluntarily provided data to the CDH Study Group Registry between 1995 and 2013.

Hospital	Country
Advocate Lutheran General Hospital	USA
Alberta Children’s Hospital	Canada
Arkansas Children’s Hospital	USA
Astrid Lindgren Children’s Hospital	Sweden
Azienda Ospedaliera Papa Giovanni XXIII	Italy
BC Children’s & Women’s Health Centre	Canada
Cardinal Glennon Children’s Hospital	USA
Carolinas Medical Center, Levine Children’s Hospital	USA
Cedars Sinai Medical Center	Los Angeles
Central Hospital Aichi Prefectural Colony	Japan
Children’s Hospital & Research Center Oakland	USA
Childrens Hospital at Skanes University Hospital	Sweden
Children’s Hospital Boston	USA
Children’s Hospital of Akron	USA
Children’s Hospital of Austin	USA
Children’s Hospital of Buffalo	USA
Children’s Hospital of Illinois	USA
Children’s Hospital of Los Angeles	USA
Children’s Hospital of Michigan	USA
Children’s Hospital of Oklahoma	USA
Children’s Hospital of Philadelphia	USA
Children’s Hospital of Wisconsin	USA
Children’s Hospital Omaha	USA
Children’s Hospitals and Clinics (Minneapolis)	USA
Children’s Memorial Hermann Hospital	USA
Children’s Mercy Hospitals & Clinics	USA
Children’s National Medical Center	USA
Cincinnati Children’s Hospital Medical Center	USA
Cleveland Clinic Foundation- Children’s Hospital	USA
Connecticut Children’s Medical Center	USA
Cook Children’s Medical Center	USA
Duke University Medical Center	USA
Emory University	USA
Freie Universitat Berlin	Germany
Georgia Health Sciences University	USA
Golisano Children’s Hospital at Strong	USA
Hasbro Children’s Hospital, Brown Medical School	USA
Helen DeVos Children’s Hospital	USA
Hershey Medical Center	USA
Hospital Clinico Universidad Católica de Chile	Chile
IRCCS Fondazione Ca’ Granda Ospedale Maggiore Policlinico	Italy
James Whitcomb Riley Children’s Hospital	Indianapolis
Juan P. Garrahan Children Hospital	Argentina
Kosair Children’s Hospital	USA
Le Bonheur Children’s Medical Center	USA
Legacy Emanuel Children’s Hospital	USA
Loma Linda University Children’s Hospital	USA
Lucile Salter Packard Children’s Hospital	USA
Massachusetts General Hospital	USA
Mattel Children’s Hospital at UCLA	USA
Mayo Clinic	USA
Medical College of Virginia	USA
Medical University of South Carolina	USA
Miami Valley Hospital	USA
National Center for Child Health and Development	Japan
NICU Health Sciences Centre	Canada
North Carolina Baptist Hospital	USA
Ospedale Pediatrico Bambino Gesù	Italy
Osaka Medical Center for Maternal and Child Health	Japan
Osaka University Graduate School of Medicine	Japan
Palmetto Health Richland	USA
Phoenix Children’s Hospital	USA
Polish Mother’s Memorial Hospital Research Institute	Poland
Primary Children’s Hospital	USA
Radboud University Nijmegen Medical Centre	The Netherlands
Rainbow Babies and Children Hospital	USA
Research Center for Obstetrics, Gynecology and Perinatology	Russia
Research Institute at Nationwide Children’s Hospital	USA
Rockford Memorial Children’s Hospital	USA
Royal Alexandra Hospital	Canada
Royal Children’s Hospital	Australia
Royal Hospital for Sick Children	Scotland
Salesi Children’s Hospital	Italy
San Diego Children’s Hospital	USA
Santa Rosa Children’s Hospital	USA
Shands Children’s Hospital/University of Florida	USA
Sophia Children’s Hospital	The Netherlands
St. Christopher’s Children’s Hospital	USA
St. Francis Children’s Hospital	USA
St. Joseph’s Hospital and Medical Center	USA
St. Louis Children’s Hospital	USA
St. Paul Campus Children’s Minneapolis	USA
Stollery Children’s Hospital	Canada
Sydney Children’s Hospital	Australia
T.C. Thompson Hospital	USA
Texas Children’s Hospital	USA
The Children’s Hospital of Alabama	USA
The Children’s Hospital of Pittsburgh of UPMC	USA
The Hospital for Sick Children	Canada
Tulane University Hospital	USA
UNC School of Medicine	USA
Universitatsklinikum Mannheim gGmbH	Germany
University Hospital Gasthuisberg	Belgium
University Malaya Medical Centre	Malaysia
University of California San Diego	USA
University of Chicago	USA
University of Kentucky Medical Center	USA
University of Michigan, C.S. Mott Children’s Hospital	USA
University of Mississippi Medical Center	USA
University of Nebraska Medical Center	USA
University of New Mexico Children’s Hospital	USA
University of Padua	Italy
University of Puerto Rico Medical Center	USA
University of Texas Medical Branch at Galveston	USA
University of Virginia Medical School	USA
Vanderbilt Children’s Hospital	USA
Vladivostok State Medical University	Russia
Wilford Hall USAF Medical Center	USA
Winnie Palmer Hospital for Women & Babies	USA
Yale New Haven Children’s Hospital	USA
